# Alcohol consumption at addiction congresses and specialized trainings: evidence, resistance and institutional change

**DOI:** 10.1192/j.eurpsy.2026.12012

**Published:** 2026-07-31

**Authors:** F. Calvo

**Affiliations:** Departament de Pedagogia, Universitat de Girona, Girona, Spain

## Abstract

**Introduction:**

The presence of alcohol at congresses and training events on addictions normalizes its use in spaces that should instead be protective and consistent with evidence on population-level harms. Moreover, the notion of “responsible drinking” is ambiguous and largely functional to industry interests.

**Objectives:**

To describe alcohol availability and management at academic/professional events on addictions.

To analyze institutional responses to proposals for eliminating alcohol from official events.

To explore ethical and clinical implications for psychiatry and public health.

**Methods:**

Qualitative autoethnographic design. Field diary, participant observation, and analysis of public/organizational materials were conducted at: two Socidrogalcohol congresses (2021, 2024), Lisbon Addictions (2022), ISSUP/ICUDDR (2024), and social events of a specialized master’s program. Correspondence with committees and meeting minutes/decisions were also reviewed when available. Study period: 2021–2024. Analytical framework: normalization of consumption and professional modeling.

**Results:**

- High availability of alcohol in several events (including “open bar” at some), often justified as “gastronomic” or under the label of “responsible drinking,” perceived as strategically ambiguous.
- International variability: some congresses excluded alcohol from official events for reasons of participant respect and risk reduction, while others offered wide alcohol availability.
- Contextual effects observed: disinhibition and safety/ethical dilemmas; potential triggering role for people in recovery; contradictory messages to the professional community.
- Institutional change: majority vote in a Spanish scientific society to stop serving alcohol at official events, though with both support and resistance.

**Conclusions:**

In addiction-specialized contexts, a gap persists between preventive discourse and social practice. Eliminating alcohol from official events, systematically offering alternatives, and aligning institutional policies with scientific evidence are feasible measures consistent with psychiatric and public health practice. The “responsible drinking” label should not guide policy in these contexts due to its ambiguity and potential to normalize consumption.

**Disclosure of Interest:**

None Declared

